# Tibial tuberosity osteotomy and medial patellofemoral ligament reconstruction for patella dislocation following total knee arthroplasty: A double fixation technique

**DOI:** 10.1051/sicotj/2022023

**Published:** 2022-06-14

**Authors:** Jobe Shatrov, Antoine Colas, Gaspard Fournier, Cécile Batailler, Elvire Servien, Sébastien Lustig

**Affiliations:** 1 Orthopaedics Surgery and Sports Medicine Department, FIFA Medical Center of Excellence, Croix-Rousse Hospital, Lyon University Hospital 103 Grande Rue de la Croix Rousse 69004 Lyon France; 2 Univ Lyon, Claude Bernard Lyon 1 University, IFSTTAR, LBMC UMR_T9406 69622 Lyon France; 3 Sydney Orthopaedic Research Institute (SORI), Orthopaedics 500 Pacific Hwy St Leonards NSW Australia

**Keywords:** Medial patellofemoral ligament reconstruction, Total knee arthroplasty, Patella instability, Knee, Knee arthroplasty

## Abstract

*Introduction*: Patella instability post total knee arthroplasty (TKA) is a rare complication. Tibial tubercle osteotomy (TTO) with medial patellofemoral ligament reconstruction (MPFLr) has not been well described for this indication. This paper describes a surgical technique to address the unique challenges faced when performing TTO and MPFLr in the prosthetic knee. *Technique*: This technique and video describe a TTO and MPFLr via an extensile incision and medial sub-vastus approach. A 6 cm long TTO is performed, if indicated, to medialise the extensor mechanism up to 1 cm and fixed with ×2 4.5 mm cortical screws. For the MPFLr, a quadriceps tendon autograft is utilized, with the natural insertion to the superior pole of the patella being left undisturbed. The graft is first attached with an interference screw and then reinforced with an endobutton to provide crucial cortical fixation to overcome the problem of low bone mineral density encountered in this area of the femur following TKA. *Results*: Five patients underwent MPFLr using the described technique. No failures or recurrence of instability occurred at the last follow-up. Pre-operative mean patella tilt and shift were 44° and 3.5 cm, respectively. Post-operatively, mean tilt and shift were 4.1° and 0.4 cm, respectively. There was one wound dehiscence requiring surgical debridement and closure. *Conclusion*: This paper describes a surgical technique to perform a TTO and MPFLr for patella instability post-TKA. The described method highlights key adaptations to address the unique challenges in this patient population.

## Introduction

Patellofemoral (PF) complications are the most common non-infective cause of revision following total knee arthroplasty (TKA) [1, 2]. Of all PFJ problems following TKA, instability is one of the most uncommon but difficult to manage scenarios the surgeon faces.

Tibial tubercle osteotomy (TTO) and reconstruction of the medial patellofemoral ligament (MPFL) have been used successfully as a surgical treatment for lateral patella instability in the native knee [3–5]. Typically, patients are young, participate in sports, have not had previous surgery, and have good bone quality [6]. The use of MPFLr and techniques to address some of the unique challenges faced in this scenario is not well described.

This study aimed to illustrate and describe a surgical technique to transpose the tibial-tuberosity medially and reconstruct the MPFL for patella instability post-TKA using double fixation using a quadriceps tendon autograft.

## Surgical technique

The surgical technique is shown in Video 1 (see Section “Data availability”).

Indications for Tibial-Tubercle-Osteotomy

### Examination, positioning, and setup

**Figure 1:**
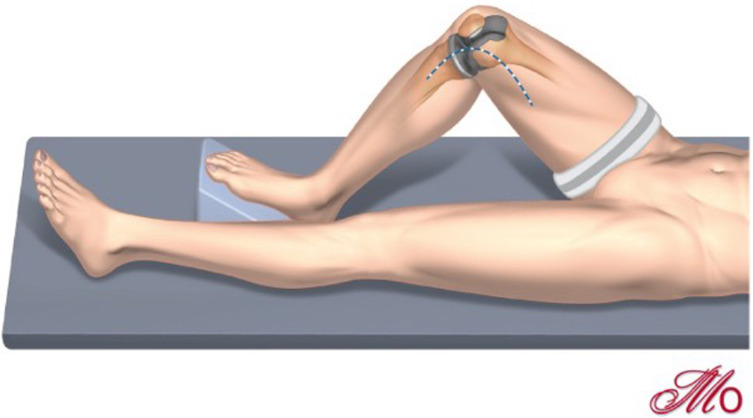
Positioning and incision planning. Positioning the limb at 90° flexion will facilitate the exposure. The medial-sub vastus approach is performed, which aids later graft passage, allows access to the joint if arthrotomy is required, and facilitates a TTO if necessary. Skin incision will require utilizations of scars, however, it should be kept in mind the deeper dissection lies medial – depicted here by the blue dotted line.

An examination under anesthesia is performed to assess the knee range of motion, coronal, and sagittal balancing in full extension and 90° flexion, presence of a J-sign, the mobility and reducibility of the patella, and the range of instability. Patella instability post-TKA may be a sign of imbalance, particularly in flexion, and a careful assessment of the prosthesis to check for this should be performed. The quadriceps muscle belly, and tendon should be palpated for any defects, which, if present, may require an alternative graft choice to be required to the one described in this technique. Positioning is shown in Figure 1.

### Incision and exposure

A midline incision is preferred. In the case of multiple incisions, the more lateral approach is used to preserve blood supply to the skin flap. The length of the incision is dictated by the graft harvest, as well as any planned additional procedures such as a TTO. Typically, a length 10 cm superior to the patella is required for graft harvesting.

**Figure 2:**
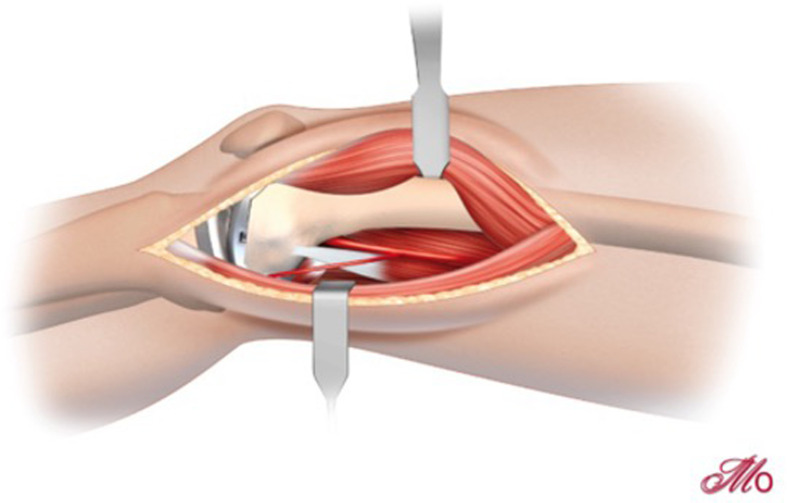
Deep dissectio. A blunt retractor is placed beneath the muscle belly of the VMO, elevating it away from the medial side of the distal femur. The descending genicular vessels, accompanying veins, and vessels to vastus medialis should be identified and ligated or cauterized to avoid a post-operative hematoma.

Access to the medial aspect of the femur and the joint, if necessary, is performed via a medial sub-vastus approach (Figure 1). Briefly, the aponeurosis of the muscle is incised in flexion, and the plane between muscle and fascia is developed by blunt dissection until the bone is reached. The leg is then positioned in extension, and a blunt retractor is placed between the muscle and capsule to allow the muscle to be retracted laterally. Particular attention should be paid to cauterizing a leash of vessels running along the bone surface deep to the muscle belly running in an oblique fashion towards the midline approximately 5–6 cm from the medial epicondyle (Figure 2).

### Tibial-tubercle-osteotomy

If TTO is to be performed, it is essential to do so before any soft tissue reconstruction because it will affect the length of the reconstruction.

A TTO to treat patella dislocation following TKA should be performed for the following indications:

1. Quadriceps shortening, for example, chronic or irreducible patella dislocation
2. J-Sign during examination under anesthesia
3. Severe patella Baja

TTO is performed using an oscillating saw to create an osteotomy 6 cm in length, 1.5 cm deep proximally, tapered distally, and hinged open from the medial side. The osteotomy is fixed using two 3.5 mm cortical screws. Medialization is performed up to a maximum of 10 mm or until the J-sign and proximalization are added in cases of quadriceps shortening or severe patella baja. In cases requiring proximalization, the osteotomy is adapted to preserve a 1 cm bone bridge to avoid conflict with the tibial tray.

### Graft harvest and preparation

**Figure 3:**
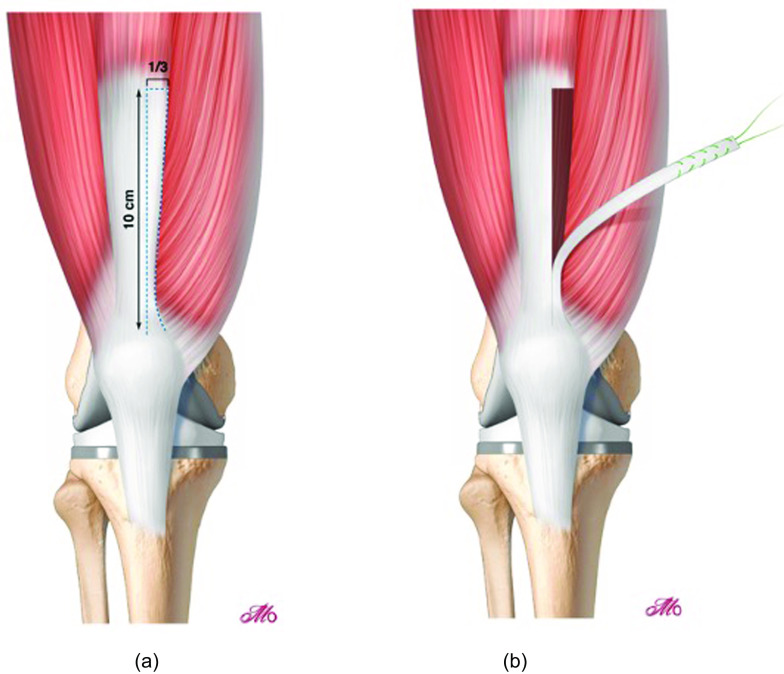
(A) Graft harvest planning: 10 cm of the quadriceps tendon is performed, taking the medial 1/3 of the quadriceps tendon and whipstitching with a non-resorbable suture. The graft is left attached to the patella distally. A thin cuff of the tendon is left attached to the VMO to facilitate later closure of the defect. (B) Graft harvest: The quadriceps tendon has 3 and sometimes 4 layers that distally fuse but can still be defined more easily proximally where they become more distinct. The graft harvest should include the full thickness of the tendon.

**Figure 4:**
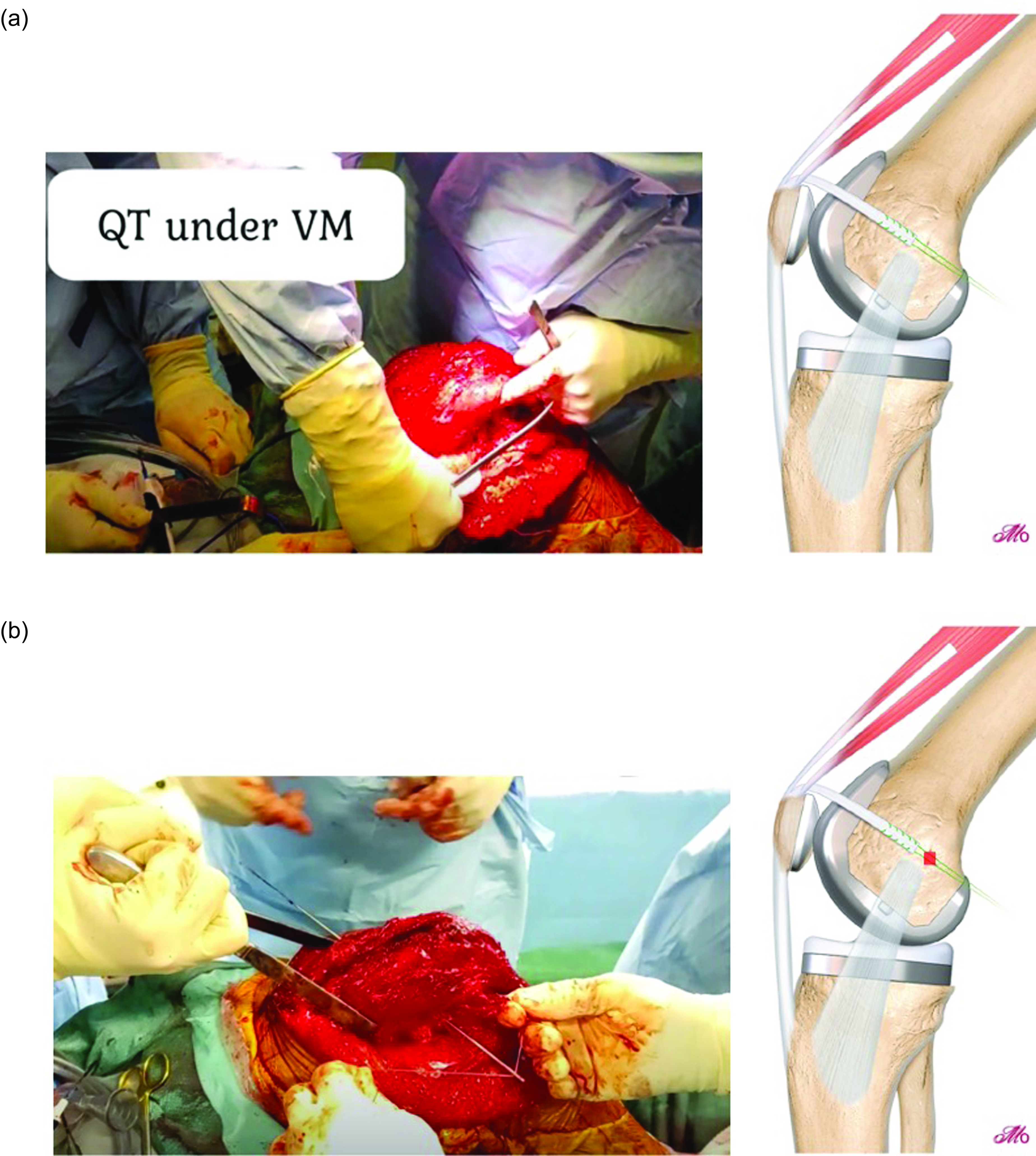
(A) Graft passage: The graft is passed beneath vastus medialis muscle and is ready to pass into the femoral tunnel. (B) Femoral tunnel: The femoral tunnel is drilled starting midway between the adductor tubercle and the medial femoral epicondyle. Tunnel trajectory should aim approximately 30° proximally in order for the tunnel to exit through cortical bone on the lateral side of the distal femur rather than through cancellous bone. The native MPFL footprint (depicted here by the red square) is approximately halfway between the adductor tubercle and medial epicondyle.

The graft is left attached to its insertion onto the patella. Care is taken to expose the tendon to its medial and lateral edges, following which 10 cm in length (Figure 3A) is harvested. A length of 10 cm is preferred, allowing for 6–7 cm length from the patella to the tunnel origin, and a minimum of 2, but preferably 3 cm of the graft to be in the tunnel. The tendon harvested is divided proximally and left attached to the patella distally (Figure 3B). The medial third of the tendon is preferred as this does require the graft to be tunneled under or over the remaining quadriceps tendon. Once harvested, the free end of the tendon is whip stitched and passed beneath the muscle belly of vastus medialis (Figure 4A).

### Tunnel positioning

A femoral bone tunnel is created with the starting point at its anatomical origin. This has previously been described as arising from the medial upper 2/3 of the patella and attaching to the femur midway between the adductor tubercle and the medial femoral epicondyle (Figure 4B) [7]. Typically, we find that an intra-operative X-ray to confirm tunnel positioning is not required due to the exposure, however, it can be performed at this point.

Before drilling the tunnel, a guidewire is inserted, the free ends of the whip stitch in the graft are wrapped around the guidewire, and the knee is ranged through an arc of 90° flexion to check for graft isometry. Adjustments are made depending on the isometry of the graft and the stability of the patella, for example, if the graft is loose in flexion, the position is moved posteriorly, too tight, and moves anteriorly, etc.

Once tunnel position has been deemed satisfactory, the guidewire is advanced through the contralateral cortex, aiming slightly proximally to avoid the prosthesis (Figure 4B). Next, a 4.5 mm cannulated drill is advanced through both cortices, and following this, a 6 mm cannulated drill is used to create a socket for the graft 30 mm in length. Next, the ends of the suture attached to the graft are passed through the islet of the guidewire, which is used to shuttle the graft through to the lateral side.

### Graft tension and fixation

Graft tensioning is performed between 30 and 45° of flexion. Tension is determined by achieving the goal of the surgery, which is to correct tilt and allow 100° of flexion without subluxation or dislocation of the patella. Typically, more tension is required in the graft than what would be performed for a primary MPFL reconstruction in a younger active patient with a native knee joint.

**Figure 5:**
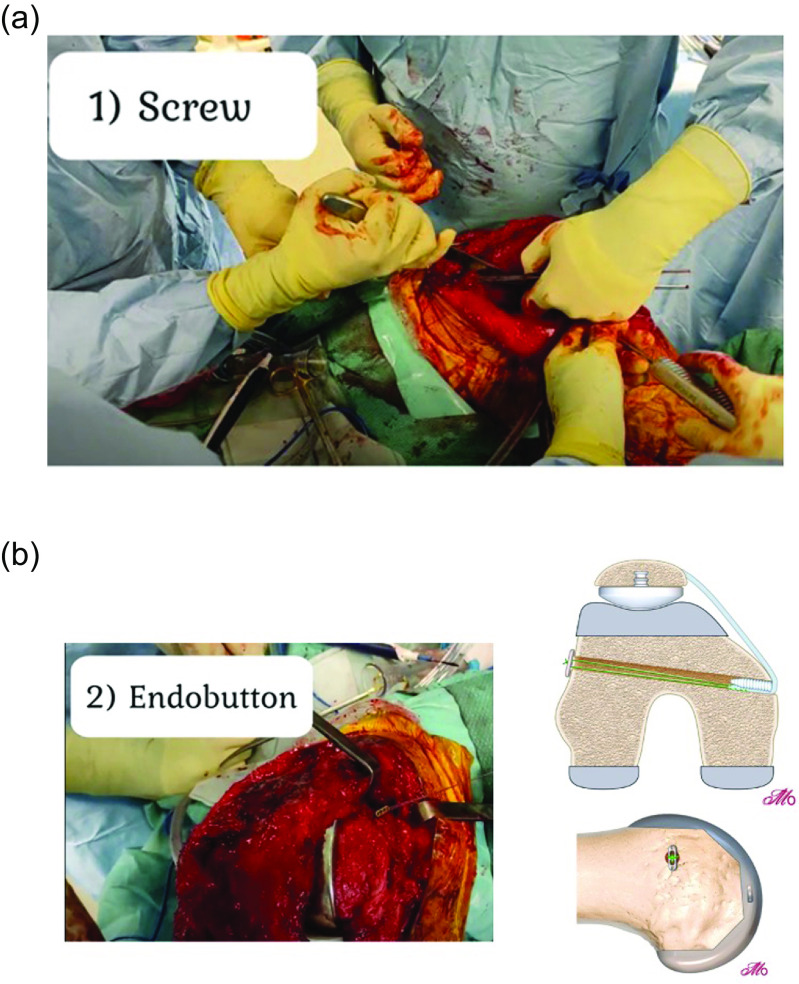
(A) Graft fixation: The graft is fixed first with an interference screw. Note tensioning is performed at 30–45°. (B) Double fixation: An endobutton is tied flush with the lateral femoral cortex using the free ends of the ship stitch from the graft. This is considered the critical step, and the button should be tied flush onto cortical bone.

Graft fixation is achieved first using an interference screw (Figure 5A). Next, an Endobutton CL (Smith & Nephew Endoscopy, Andover, MA) is loaded onto the two non-absorbable suture tails and tied down (Figure 5B). An important step of the cortical fixation is to ensure the button sits flush with the lateral cortex and no soft tissue interposition is present between the button and the cortical bone to avoid a springing effect on the graft or subsidence with time. Table 1 summarizes the technical pearls and pitfalls of this procedure.

**Table 1:** Pearls and pitfalls.

Pearls
Perform an EUA prior to skin incision to confirm the range at which the patella is unstable.
A medial sub-vastus approach is an ideal exposure as it facilitates graft passage and can also be used to access the joint if necessary.
Using quadriceps tendon autograft allows the graft to remain attached to the patella through its natural attachment and avoids any fixation into the patella being required.
Leaving a thin cuff myotendinous junction attached to the vastus medialis during harvest facilitates later closure of the defect and creates a small “reefing” effect by advancing the muscle slightly, which will add to the stability of the reconstruction.
Graft tensioning should be performed at 30–45° flexion.
Tendency for tensioning the graft should be to increase tightness, rather than avoiding over-tensioning like in native patellae MPFL reconstruction.
Tensioning is performed to correct dislocation, J-tracking, and tilt. Pay attention to the range of flexion that demonstrated patella sub-luxation in the pre-operative EUA to confirm adequate correction and graft tensioning.
Add cortical fixation to the graft after screw insertion and ensure the button is seated flush on the lateral cortex to avoid the “springing” effect of soft tissue interposition.
Quadriceps tendon graft may be bulky, and to facilitate passage into the tunnel, tubularization of the tip of the graft may be beneficial.
Pitfalls
Not performing the TTO if it is indicated prior to the soft tissue reconstruction.
Failure to plan sufficient graft length may compromise the isometry of the graft and the stability of the graft fixation.
Reliance on interference fixation in the supracondylar region of the femur may lead to graft slippage and recurrence of instability due to low BMD in this region post-TKA.
Under tension, the graft is likely to lead to residual instability.
If any additional procedures to the patella are planned, they should be performed first, as the effect on graft tension is unpredictable and, if done following reconstruction, may lead to insufficient graft tension.

### Rehabilitation protocol

Post-operatively the patient is placed into a range-of-motion knee brace that allows a range of movement from 0 to 90° of flexion. The patient can fully-weight bear with the brace locked in full extension. Follow-up consultation at 6 and 12 weeks is performed with X-rays to look at patella height, tilt, and translation. If a TTO is performed, X-rays are taken until the radiographic union is achieved, which is usually 12 weeks following surgery.

All procedures performed were in accordance with the ethical standards of the institutional and national research committee and with the 1964 Declaration of Helsinki and its later amendments or comparable ethical standards.

## Results

Between May 2019 and January 2021, five patients with a mean age of 73.4 years underwent MPFLr using the double fixation technique described for patella instability post-TKA. Patient baseline characteristics are presented in Table 2.

**Table 2:** Patient characteristics.

Case no.	Age	Gender	Aetiology of TKA	Patella	BMI	ASA	Previous knee surgeries	PS	CDI	PT	ROM	HKA	Femoral rotation	Tibial rotation
1	70	F	Post-traumatic	Dislocated	31	2	4	3.72	0.7	30	0–100	181	Not available	Not available
2	71	F	Idiopathic OA	Dislocated	40	3	2	−0.5	1.26	37	0–110	178	0	6
3	83	F	Post-traumatic	Dislocated	30	3	3	4.46	0.71	40	0–85	178	1	11
4	70	M	Revision for infection	Dislocated	37	3	4	5	0.45	47	0–120	177	0	5
5	73	F	HTO	Dislocated	35	2	2	4.7	0.45	65	0–130	176	−3	10

PS = Lateral patella shift measurements (cm). Negative values indicate medial displacement and positive values indicate lateral displacement; PT = Lateral patella tilt; CDI = Caton-Deschamp Index; ROM = Range-of-motion; ASA = American Society of Anaesthesiologist grade; HKA = Hip-Knee-Angle as measure on long leg plain radiograph; BMI = Body mass index; Femoral rotation = As measured on CT scan relative to the trans-epicondylar axis. Negative values indicate external rotation, positive values indicate internal rotation; Tibial rotation = As measured on CT scan relative to the centre of the tibial-tuberosity. Positive values indicate internal rotation.

**Figure 6:**
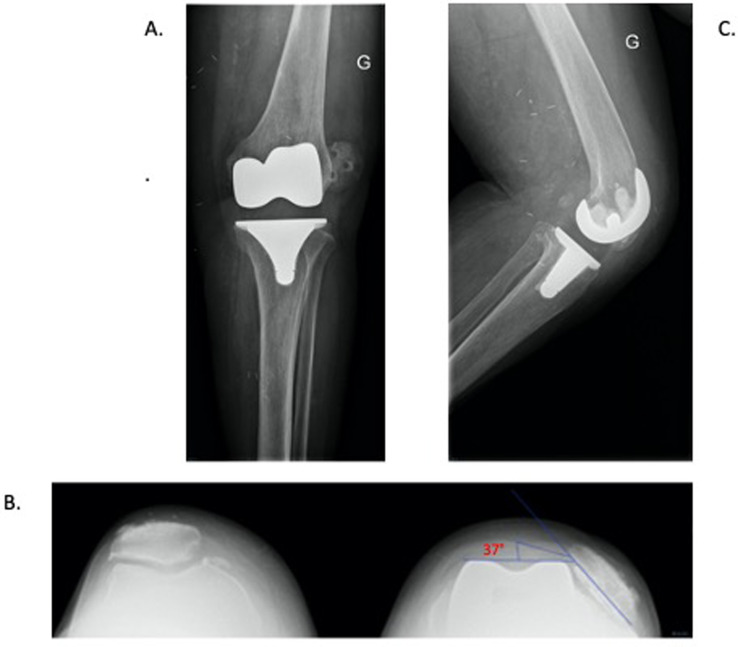
Pre-MPFL reconstruction. Pre-operative X-rays of a patient with lateral patella dislocation post total knee arthroplasty. (A) AP X-ray. The patella is seen dislocated laterally. (B) Sky-line view demonstrating patella dislocation and patella-tilt of 37°. (C) Lateral profile X-ray.

**Figure 7:**
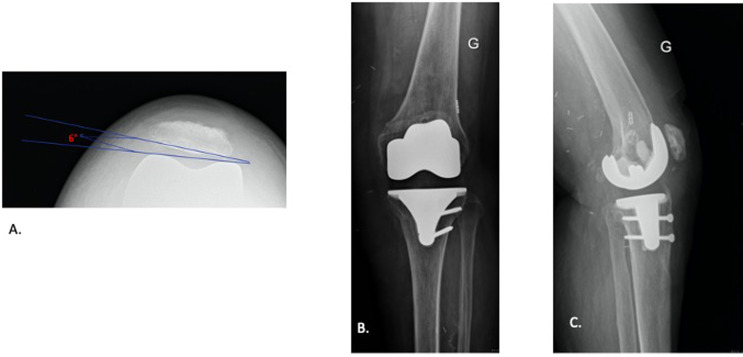
Post-MPFL reconstruction. Post-operative X-rays of the patient from Figure 1 were taken at 12 weeks post-surgery. (A) Sky-line view demonstrating patella now centered with a patella tilt of 6°. (B) AP X-ray, the endobutton can be seen sitting flush on the lateral cortex. (C) Lateral profile demonstrating the tibial-tubercle osteotomy and tunnel position. The osteotomy is not yet fully united.

Outcomes are presented in Table 3. No failures or recurrence of instability occurred at the last follow-up. Post-operatively, the mean tilt and shift were 4.1° and 0.4 cm, respectively (see for example in Figures 6 and 7). One patient experienced wound dehiscence, which required wound debridement and closure, but did not develop a deep infection. Pre- and post-operative range of motion were similar, however, most patients had a flexion range reduced by approximately 10° following the surgery.

**Table 3:** Post-operative assessment.

Case no.	MPFL graft	Additional procedure	ROM (°)	Lag	TTO union	Shift	Tilt (°)	CDI
1	QT	TTO, lateral release and patella button revised	0–95	No	6	0	1.6	0.83
2	QT	TTO	0–100	No	16	0.3	3.8	0.95
3	QT	TTO, patella resurfaced	0–100	No	12	0.6	1	1.1
4	QT	TTO	0–110	No	20	0.5	6	0.96
5	QT	TTO	0–120	Yes	12	0.7	10	0.3

PT = Patella tilt (°); PS = Patella shift (mm); ROM = Range-of-motion (°); TTO = Tibial tubercle osteotomy; CDI = Caton-Deschamps Index; QT = Quadriceps tendon autograft.

## Discussion

Patella instability post-TKA is a rare but difficult complication to manage. TTO and MPFLr have been described in the management of native patella instability, however, several important differences exist in the arthroplasty patient population that requires adaptation of the surgical technique. A crucial aspect of the MPFLr technique is the addition of cortical fixation to address the low bone density in the region of the femoral tunnel.

Surgical management of patella instability after TKA broadly follows one of two pathways [8]. Either revision of components deemed to be malpositioned [9] or patella stabilization with soft-tissue, bony or combined procedures [8, 10, 11]. Implant positioning limits have not been well defined, and revision of well-fixed implants is a morbid procedure associated with bone loss and soft tissue damage that often requires the use of highly constrained implants that have twice the failure rate of primary implants after 10 years [12]. Furthermore, certain implant design features are prone to patellofemoral instability, making them more sensitive to malpositioning [10]. For example, femoral components with symmetrical shallow trochlear grooves are predisposed to patellar subluxation [13, 14]. Alternative approaches to major revision have been advocated, such as a TTO combined with a lateral release [11]. However, the presence of a stem increases the difficulty of this procedure [8] and also fails to address the soft tissue imbalance invariably created by the stretching and tearing of medial-sided soft tissue structures that occur when the patella dislocates laterally. Vast medialis advancement with lateral release has been described in this setting [15], albeit in a single case study with 8 months follow-up [16]. Additionally, MPFLr has better outcomes for native patella instability compared to VMO advancement, and the plication of tissue that is scarred from multiple previous surgeries and trauma is difficult and may not provide reliable results. MPLFr in this setting has been described using hamstrings [17], however graft attachment to the resurfaced patella is challenging, hence we prefer a graft that utilizes the medial 1/3 of the quadriceps tendon and has been described previously by Van Gennip et al. [18].

Double fixation is a critical step of the MPLFr procedure as it addresses the poor bone quality that can affect graft stability and is frequently encountered in the supracondylar region of the femur following TKA. Bone mineral density (BMD) in the supracondylar region of the femur following TKA has decreased by 20–45% following TKA [19, 20]. Most studies that have reported on MPFLr to treat patella instability post-TKA describe an interference fixation for the graft [17, 18], which relies on compressing the soft tissue against bone to prevent slippage. Load to failure using interference screw fixation is directly related to BMD due to graft slippage at significantly lower forces than alternative methods during biomechanical testing [21].

## Conclusion

This paper describes a surgical technique to perform a TTO and MPFLr for patella instability post-TKA. The described method highlights key adaptations to address the unique challenges in this patient population.

## Data Availability

The video is available at the following URL: https://www.youtube.com/watch?v=WGAB5s5nnao.
